# GLAD4U: deriving and prioritizing gene lists from PubMed literature

**DOI:** 10.1186/1471-2164-13-S8-S20

**Published:** 2012-12-17

**Authors:** Jérôme Jourquin, Dexter Duncan, Zhiao Shi, Bing Zhang

**Affiliations:** 1 Department of Biomedical Informatics, Vanderbilt University School of Medicine, 400 Eskind Biomedical Library, 2209 Garland Avenue, Nashville, TN 37232, USA; 2 Department of Cancer Biology, Vanderbilt University School of Medicine, 2220 Pierce Avenue, PRB771, Nashville, TN 37232, USA; 3 Advanced Computing Center for Research & Education, Vanderbilt University, Nashville, TN 37240, USA; 4 Department of Electrical Engineering and Computer Science, Vanderbilt University, Nashville, TN 37240, USA

## Abstract

**Background:**

Answering questions such as "Which genes are related to breast cancer?" usually requires retrieving relevant publications through the PubMed search engine, reading these publications, and creating gene lists. This process is not only time-consuming, but also prone to errors.

**Results:**

We report GLAD4U (Gene List Automatically Derived For You), a new, free web-based gene retrieval and prioritization tool. GLAD4U takes advantage of existing resources of the NCBI to ensure computational efficiency. The quality of gene lists created by GLAD4U for three Gene Ontology (GO) terms and three disease terms was assessed using corresponding "gold standard" lists curated in public databases. For all queries, GLAD4U gene lists showed very high recall but low precision, leading to low F-measure. As a comparison, EBIMed's recall was consistently lower than GLAD4U, but its precision was higher. To present the most relevant genes at the top of a list, we studied two prioritization methods based on publication count and the hypergeometric test, and compared the ranked lists and those generated by EBIMed to the gold standards. Both GLAD4U methods outperformed EBIMed for all queries based on a variety of quality metrics. Moreover, the hypergeometric method allowed for a better performance by thresholding genes with low scores. In addition, manual examination suggests that many false-positives could be explained by the incompleteness of the gold standards. The GLAD4U user interface accepts any valid queries for PubMed, and its output page displays the ranked gene list and information associated with each gene, chronologically-ordered supporting publications, along with a summary of the run and links for file export and functional enrichment and protein interaction network analysis.

**Conclusions:**

GLAD4U has a high overall recall. Although precision is generally low, the prioritization methods successfully rank truly relevant genes at the top of the lists to facilitate efficient browsing. GLAD4U is simple to use, and its interface can be found at: http://bioinfo.vanderbilt.edu/glad4u.

## Background

The physical development and phenotype of organisms can be thought of as a product of genes interacting with each other and with the environment. Therefore, it is common for a scientist to ask questions like "Which genes are related to breast cancer?", "Which genes are involved in embryonic development?", and "Which genes are functionally related to TP53?"

The current answers to these questions are primarily contained in the articles indexed in the MEDLINE database. Traditionally, answering these questions requires individuals to retrieve relevant publications through the PubMed search engine and then to create gene lists by manually extracting gene-centered information from retrieved literature. This process is not only time-consuming, but also prone to errors. First, it is difficult to ascertain that all relevant literature is processed. Second, it is unlikely that all relationships in a publication will be detected. Third, individual researchers tend to extrapolate based on domain knowledge.

Over the past decade, bioinformatics approaches have been developed to address this issue. One of the most successful projects in this area is the Gene Ontology (GO) project [1]. GO produces a structured, precisely defined, and controlled vocabulary (i.e., GO terms) for describing the roles of genes and gene products in different species. Genes are associated with GO terms through manual curation as well as computational inference. A researcher can now go to the GO website [2] to get a list of genes related to a GO term of interest. However, as the GO vocabulary only describes gene products in terms of their associated biological processes, cellular components and molecular functions, users are limited by questions linked to this limited vocabulary. Moreover, processes, functions or components that are unique to diseases, such as oncogenesis, are not included in GO because causing cancer is not the normal function of any gene.

A useful resource specifically designed for disease studies is the Online Mendelian Inheritance in Man (OMIM [3]) project. OMIM is a comprehensive, authoritative, and timely compendium of human genes and genetic phenotypes. It contains information on all known Mendelian disorders. However, information on complex diseases such as cancer and diabetes is lacking in OMIM.

In addition to manual curation, text mining tools have been developed to assist gene list creation [4]. As an example, EBIMed [5,6] combines text mining with co-occurrence-based analysis to generate a prioritized list of genes for a user-provided query. Specifically, EBIMed collects MEDLINE records and available full text documents for a user-provided query, identifies protein names, drugs, species, or GO terms in the documents, and prioritizes genes/proteins based on the number of co-occurrences of the different pairs (protein/protein, protein/drug, protein/species, protein/GO term) in the sentences of the documents in which they appear. EBIMed and similar tools, such as FACTA [7] and SciMiner [8], provide more flexible ways to create gene lists that are not limited to certain aspects of biology. Nevertheless, they usually require heavy computation, and the relevance of the resulted gene lists to the input queries has not been systematically evaluated.

Here, we report GLAD4U (Gene List Automatically Derived For You), a new web-based gene retrieval and prioritization tool. GLAD4U takes advantage of existing resources at the National Center for Biotechnology Information (NCBI) to ensure computational efficiency. It provides a simple user interface that facilitates intuitive usage and interpretation of results. The quality of gene lists created by GLAD4U is assessed using corresponding "gold standard" lists curated in GO, GAD (Genetic Association Database [9]), and OMIM. The performance of GLAD4U is also compared with EBIMed.

## Results

### Overall quality of the retrieved gene lists

GLAD4U relies on the NCBI eSearch API to find publications related to a user query and on the gene-to-publication link table to identify genes from the retrieved publications. We used three GO biological process terms (apoptosis, cell adhesion and DNA repair) and three disease terms (hypertension, obesity and schizophrenia) as queries to evaluate the overall quality of the retrieved gene lists. For each query, using a corresponding gene list curated by GO or GAD/OMIM as a gold standard, we calculated the precision, recall and F-measure of the retrieved gene list. As shown in Table 1, gene lists retrieved for all queries showed very high recall (0.90±0.03 for GO terms and 0.96±0.05 for disease terms). In contrast to the high recall, the precision was generally low (0.16±0.04 for GO terms and 0.06±0.02 for disease terms), leading to low F-measures (0.27±0.05 for GO terms and 0.12±0.03 for disease terms). EBIMed's recall is consistently lower than GLAD4U (0.47±0.15 for GO terms and 0.44±0.11 for disease terms). However, its precision is higher than GLAD4U (0.20±0.05 for GO terms and 0.16±0.04 for disease terms), resulting in better F-measures (0.27±0.03 for GO terms and 0.23±0.04 for disease terms).

**Table 1:** Overall quality of the retrieved gene lists

Query	GO/ MIM gene count	GLAD4U gene count	EBIMed gene count		GLAD4U	EBIMed
**Apoptosis**	1039	6037 (958)	1469 (387)	Precision	0.1587	0.2634

		[195715]	[10000]	Recall	0.9220	0.3725

				F-measure	0.2708	0.3086

**Cell adhesion**	785	4195 (691)	1725 (305)	Precision	0.1647	0.1769

		[125144]	[10000]	Recall	0.8802	0.3885

				F-measure	0.2775	0.2431

**DNA repair**	282	2476 (263)	1100 (180)	Precision	0.1062	0.1636

		[60952]	[10000]	Recall	0.9326	0.6383

				F-measure	0.1907	0.2605

**Hypertension**	87	2046 (77)	135 (27)	Precision	0.0376	0.2000

		[323818]	[10000]	Recall	0.8851	0.3103

				F-measure	0.0721	0.2432

**Obesity**	111	1778 (110)	350 (59)	Precision	0.0619	0.1686

		[141615]	[10000]	Recall	0.9910	0.5315

				F-measure	0.1165	0.2560

**Schizophrenia**	94	1725 (90)	382 (44)	Precision	0.0522	0.1152

		[91194]	[10000]	Recall	0.9574	0.4681

				F-measure	0.0990	0.1849

Numbers in parentheses indicate the number of genes overlapping between the GLAD4U or EBIMed lists and the corresponding gold standard, numbers in square brackets indicate the number of publications retrieved by the query.

The low precision of GLAD4U may be partially attributed to the incompleteness of the annotation in GO and GAD/OMIM. However, it is likely that the original gene lists include many irrelevant genes. In this case, a prioritization step that ranks truly relevant genes at the top of a list would certainly facilitate efficient browsing.

### Performance of the prioritization methods

We studied the performance of two methods to prioritize the gene lists. The first, "GLAD4U Counts", is based solely on the number of supporting publications as commonly implemented in other software [10,11]. The second, "GLAD4U Hypergeometric", is proposed in this study, which is based on the Hypergeometric test (see the Methods section for details). We used the above mentioned three GO terms and three disease terms as queries to evaluate the performance of our prioritization methods. We also included the prioritized gene lists returned by EBIMed for comparison.

**Figure 1:**
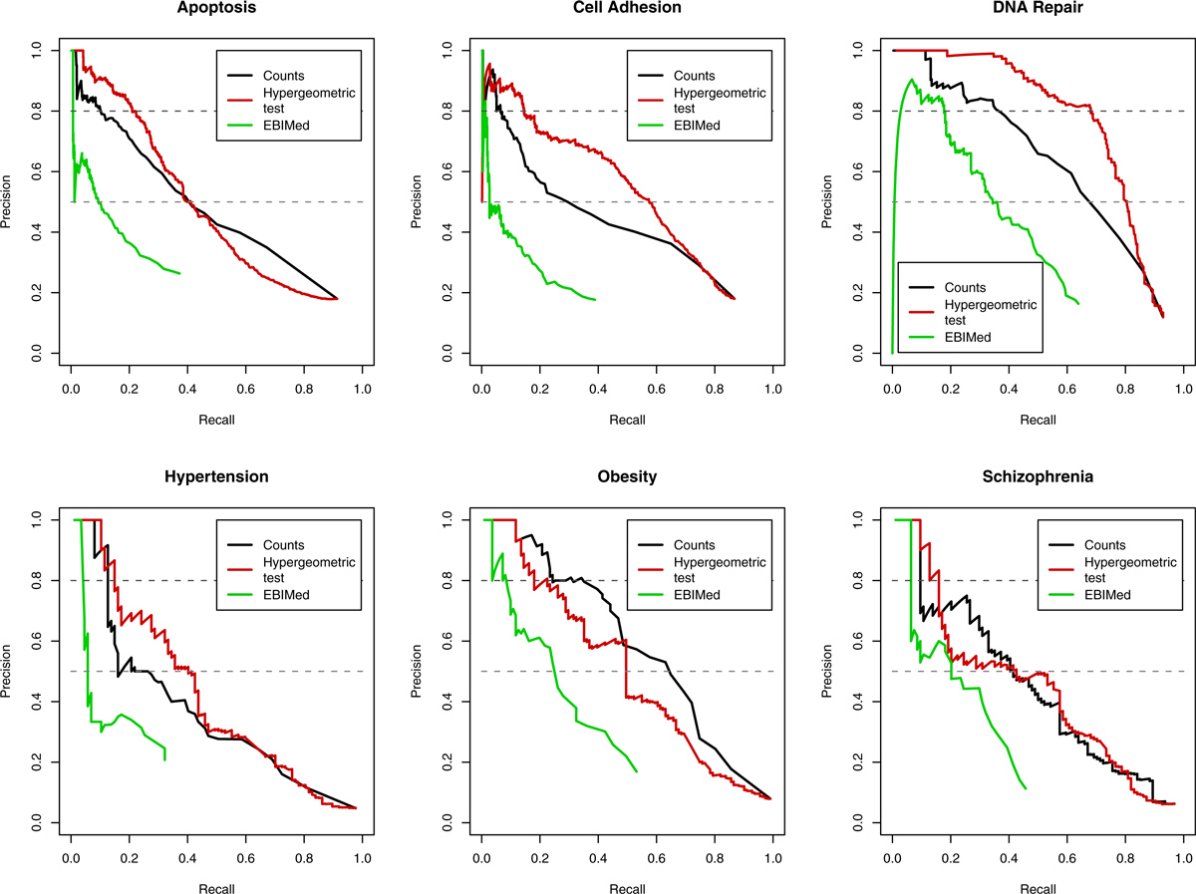
**Precision/recall curves for different prioritization methods**. Precision/recall curves for GLAD4U Counts, GLAD4U Hypergeometric and EBIMed are colored in black, red, and green, respectively. Dashed lines correspond to the precision levels of 0.8 and 0.5.

Figure 1 depicts the precision/recall curves from this comparative evaluation. For all queries, based on manual inspection of the curves, both GLAD4U Counts and GLAD4U Hypergeometric outperformed EBIMed, especially at the high precision range. Between the two GLAD4U methods, the Hypergeometric method performed better than the Counts method for GO term queries, while their performances were comparable for disease term queries. The superior overall performance of the two GLAD4U methods over EBIMed was further evaluated by computing AP, a quantitative measure of quality across all recall levels (Table 2). In this analysis, GLAD4U Counts and Hypergeometric methods scored better than EBIMed (0.48±0.10, 0.52±0.12 and 0.21±0.09, respectively), with GLAD4U Hypergeometric performing the best (Table 2).

**Table 2:** Comparison of different prioritization methods

	Apoptosis	Cell Adhesion	DNA Repair	Hypertension	Obesity	Schizophrenia
**GLAD4U Counts**						

AP	0.4939	0.4611	0.6670	0.3947	0.5698	0.4601

Precision at *k *= 50	0.8600	0.8000	0.8800	0.4800	0.7800	0.5400

Precision at *k *= 100	0.8300	0.7300	0.8100	0.3800	0.5500	0.4200

**GLAD4U Hypergeometric**						

AP	0.4942	0.5723	0.8139	0.4564	0.4782	0.4280

Precision at *k *= 50	0.9400	0.9000	1.0000	0.5800	0.6200	0.4800

Precision at *k *= 100	0.9000	0.8500	0.9700	0.3900	0.5200	0.4400

**EBIMed**						

AP	0.1567	0.1256	0.3517	0.1336	0.2673	0.2318

Precision at *k *= 50	0.6200	0.4800	0.8400	0.3137	0.5652	0.4423

Precision at *k *= 100	0.5980	0.4848	0.6700	0.2821	0.1586	0.3200

AP: Average Precision

The precision-recall curve and the AP score factor in precision at all recall levels. For ranked gene lists, particularly in web-based applications, this may not be of interest to users. In most scenarios, what matters may be the number of relevant genes on the first page or the first several pages. "Precision at *k*" is usually used to measure precision at a fixed low level of retrieved results, e.g., the top *k *results. To this end, we calculated the precisions for the top 50 (*k *= 50) and top 100 (*k *= 100) genes for all three methods, for each query (Table 2). GLAD4U Counts and GLAD4U Hypergeometric methods maintained higher precisions for the top 50 genes compared to EBIMed (0.74±0.15, 0.77±0.20 and 0.54±0.18, respectively), as well as for the top 100 genes (0.64±0.20, 0.69±0.25 and 0.42±0.20, respectively). Although the AP-based comparison may be biased against EBIMed owing to its low overall recall, precision at 50 and 100 only focus on the top ranking genes and are not affected by the overall recall. These results suggest that GLAD4U can produce lists where relevant genes are ranked at the top.

Although precision was less than perfect even for the top ranking genes, we noticed that many false-positives could be explained by the incompleteness of the gold standards. Table 3 lists the first 10 genes--along with their first 10 supporting publications--returned by GLAD4U Hypergeometric method that were not in the corresponding gold standards for the terms "apoptosis" and "hypertension" (see additional files 1 and 2 for the complete lists of genes and supporting publications). Taking the first and last genes in the list as examples, for each term (i.e., MDM2 and ING1 for apoptosis, and REN and ACE2 for hypertension), we found strong evidence in the most recent supporting publications for linking these non-gold standard genes to the query. MDM2 has antiapoptotic effects, and its direct interaction and regulation of p53 define it as an oncogene [12-15]. It translocates to the nucleus to interact with p53 and p300, promotes cell growth by initiating p53 degradation [16,17]. Its expression is directly linked to prostate cancer patient susceptibility [18]. Inhibitor of growth family, member 1 (ING1) is involved in cell stress and DNA damage response [19-22]. Up-regulation of p33ING1b or p24ING1c, two of the three alternatively spliced transcripts of ING1 resulted in increased early apoptotic cells [23,24], probably through interactions with mdm2, p14arf, and lamin A [25,26]. This effect is dependent on the presence of functional p53 [25,27] and the H3K3me3 binding domain of IGN1 [28].

**Table 3:** First 10 genes retrieved by GLAD4U and not listed in the gold standard lists

Rank	Entrez-Gene ID (Gene symbol)	Score	PMIDs*
**Apoptosis**			

41	4193 (MDM2)	53.5212	21051655, 21051533, 20849854, 20849851, 20832750, 20822933, 20708156, 20659896, 20657550, 20644561

48	1432 (MAPK14)	40.8288	20736797, 20573801, 20558744, 20473571, 20463961, 20430109, 20393480, 20345980, 20307495, 20299663

49	4609 (MYC)	37.27.98	20714214, 20598117, 20596624, 20573831, 20564213, 20515470, 20232342, 20071475, 19996270, 19966300

54	6774 (STAT3)	35.2695	20562100, 20514402, 20507639, 20490331, 20459702, 20447714, 20213502, 20197401, 20164027, 20154216

77	5580 (PRKCD)	23.3017	20548952, 20547768, 20471435, 20093486, 19932628, 19917613, 19875824, 19833733, 19808702, 19747914

78	29126 (CD274)	23.1218	20636820, 20617899, 20587542, 20506224, 20445553, 20363965, 19916867, 19826049, 19811426, 19794071

79	142 (PARP1)	22.9308	20940411, 20665026, 20644561, 20629644, 20564216, 20453000, 20388712, 20181890, 20177052, 20072652

86	406991 (MIR21)	18.9856	20813833, 20515755, 20514462, 20447717, 20404348, 20372781, 20371612, 20346171, 20153722, 20148895

96	7295 (TXN)	16.1886	20619274, 20430109, 20298786, 20103619, 19671194, 19566940, 19328186, 19120277, 18983687, 18848838

100	3621 (ING1)	15.3784	19085961, 18836436, 18801192, 18691180, 18655775, 18533182, 18388957, 17585055, 17379210, 16607280

**Hypertension **			

10	5972 (REN)	61.9237	20925572, 20662730, 20577119, 20537141, 20429690, 20223792, 20160196, 19891555, 19673942, 19536175

12	3291 (HSD11B2)	45.7032	20597806, 19811365, 19150652, 18837962, 18573267, 18178212, 17551100, 16872738, 16778331, 16109323

14	4879 (NPPB)	36.9570	20713912, 20368210, 20350538, 20346360, 20234137, 20142024, 20113292, 20102554, 20087954, 20083731

17	4524 (MTHFR)	32.2080	21072525, 21060006, 20960113, 20852445, 20812180, 20717043, 20669348, 20637366, 20592457, 20479155

19	1401 (CRP)	31.9446	21044781, 20805569, 20733302, 20683147, 20676960, 20346360, 20339115, 20184533, 20074254, 20068351

20	4878 (NPPA)	31.6082	20577119, 20543198, 20368210, 20346360, 20137368, 19635983, 19479237, 19430483, 19346663, 19330901

21	155 (ADRB3)	28.4824	20831043, 20144152, 20044737, 19842096, 19779464, 19479237, 19131662, 18724972, 18510051, 18088254

24	1584 (CYP11B1)	24.8304	20708777, 20339375, 19820005, 19567537, 19082699, 18663314, 18294861, 17980006, 17296872, 17121536

27	59272 (ACE2)	21.4649	20831027, 20813695, 20679547, 20349406, 20160196, 20117991, 19926873, 19684612, 19289653, 19286756

29	9370 (ADIPOQ)	19.6898	21044781, 20593932, 20552610, 20528971, 20516205, 20443850, 20385503, 20376890, 20166815, 20150538

* Only the 10 most recent supporting publications are shown here. See additional files 1 and 2 for the complete list of false-positive genes and their corresponding supporting publications.

Regarding hypertension, renin (REN) is part of the renin-angiotensin system (RAS). Proteins in this system are thought as important regulators of blood pressure and are involved in the onset of hypertension [29-32]. Overexpression of REN leads to hypertension via chronic overproduction of AngII [33,34], and inhibiting the regulators of the RAS--such as REN--is a common treatment for hypertension [32]. Adiponectin (ADIPOQ) is an adipocytokine synthesized by the adipose tissue. It has been proposed as a biomarker for hypertension, as low plasma levels correlates with higher risk of hypertension [35-38], and possibly with coronary artery disease, kidney disease, left ventricular hypertrophy, and even myocardial infarction [36,39-41]. Interestingly, REN and ADIPOQ also present polymorphisms, which seem linked to therapeutic response to hypertension [31,40,42-46].

From these publications, we believe that MDM2 and IGN1 should be part of the apoptosis list, as well as REN and ADIPOQ should be part of the hypertension list. These results accentuate the incompleteness of the gold standards and suggest that GLAD4U can help in the completion of the gold standard lists.

### Thresholding score to enhance GLAD4U performance

**Figure 2:**
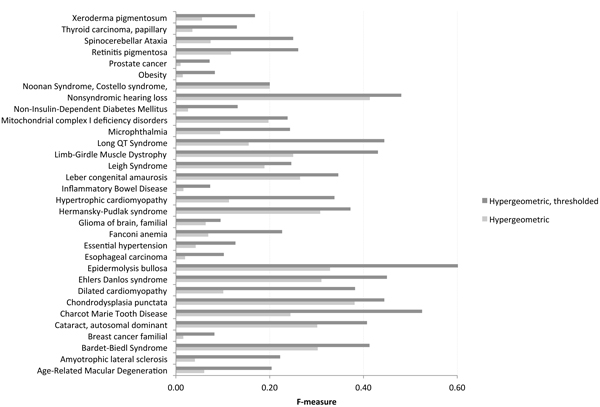
**F-measure evaluations of GLAD4U before and after thresholding**. F-measure evaluations of GLAD4U before and after thresholding, for each disease-associated gene lists. A higher F-measure indicates a better GLAD4U performance.

To evaluate whether thresholding the gene score can enhance GLAD4U performance, we acquired a broader list of disease-associated gene lists curated by Kohler et al. [47] and available from the GeneWanderer website (http://compbio.charite.de/genewanderer). We extracted 32 "disease-gene families" to use as standards for evaluating GLAD4U performance before and after thresholding. On average, GLAD4U performs 2.90-time better when genes with low prioritization scores (i.e. prioritization score < 2 or hypergeometric *p *value > 0.01) are removed, as illustrated by comparing the F-measures (Figure 2). The most increased performances were achieved for terms such as "prostate cancer", "obesity", and "amyotrophic lateral sclerosis" (folds of 7.28, 5.72, and 5.48, respectively) (see additional file 3 for the before and after F-measures, and corresponding fold-changes). The performances that least benefited from thresholding the gene list included "Noonan Syndrome, Costello syndrome, Cardiofaciocutaneous Syndrome", "Nonsyndromic hearing loss", and "Chondrodysplasia punctata" (folds of 1, 1.16, and 1.17 respectively).

### User interface

GLAD4U uses a simple query interface for users to submit their queries. Any queries that are valid in a PubMed search can be used in GLAD4U. In the query interface, users can also modify the default parameters of the application, including: search space (all species or restricted to human genes), the number of genes to present per result page, the maximum number of publications supporting each gene returned in the result page and the number of pages to build for each of the algorithm runs.

**Figure 3:**
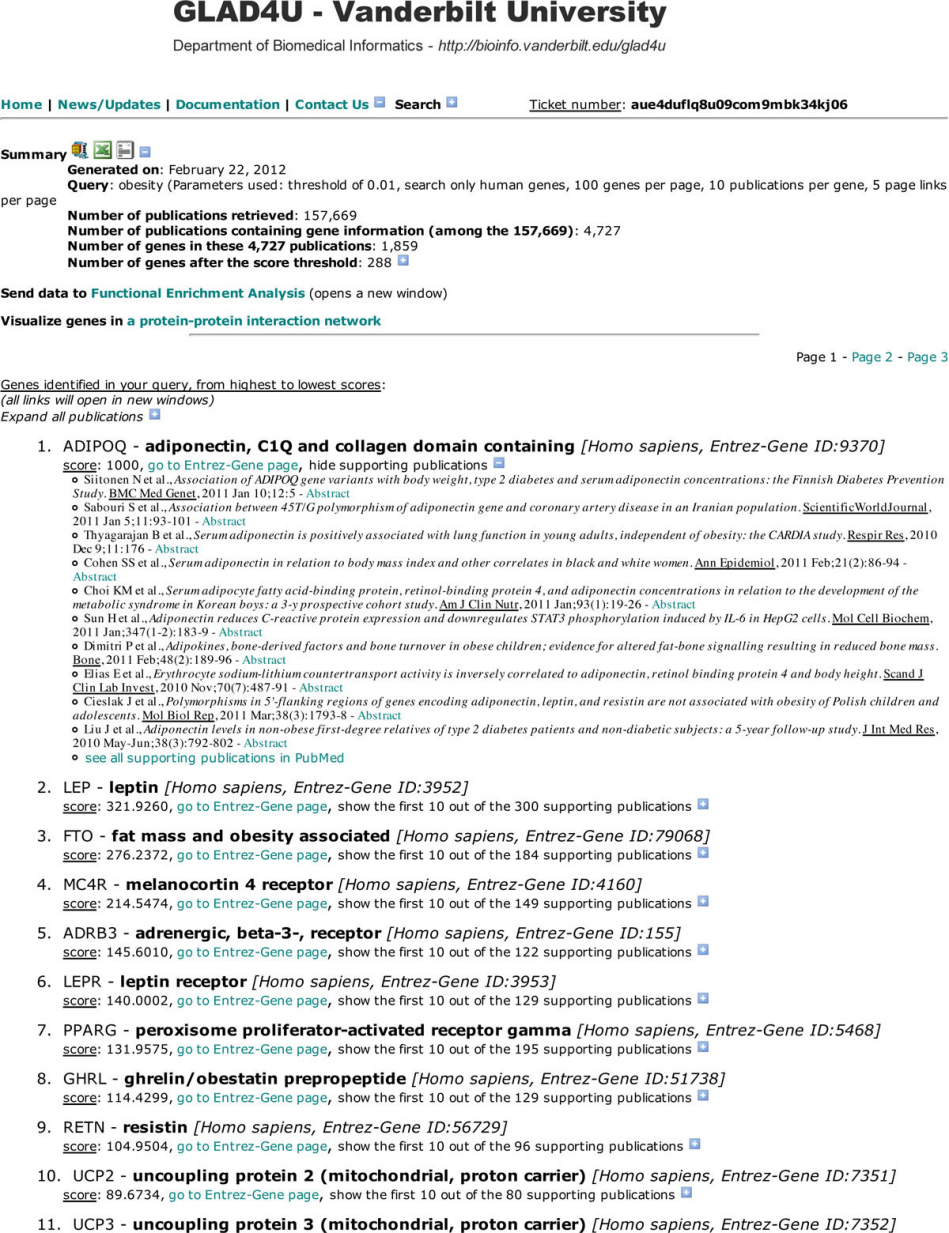
**GLAD4U output page**. A typical result page generated by a query with GLAD4U. The summary section presents the main statistics for the query, along with two hyperlinked icons to download the results as an entire archive of all pages of results ("compressed" icon), a CSV ("Excel" icon) or a text ("text" icon) file. Right below the summary, a link is available to send the results for functional enrichment analysis. In the main result section, the prioritized genes are presented. The user can click the "+" to show/hide the supporting publications, which are all hidden by default to help the read-out of the gene information. ADIPOQ gene is presented with its supporting publications as an example.

The output page displays the ranked gene list and information associated with each gene (Figure 3). As each gene is identified by an Entrez-Gene ID, we use eSummary, another NCBI's eUtility [48], to fetch annotations for the gene including name, symbol and species. Publications supporting the relationship between a gene and the query term are listed under the gene. The publications are ordered based on their PubMed IDs so that the most recent publication is listed first (see Figure 3, under the "ADIPOQ" gene description). As for genes, we use eSummary to fetch information for the publication such as title, author and journal name. Genes and publications are hyperlinked to the corresponding NCBI pages, which will--by design--open in a new window to avoid disrupting the result page.

At the top of the output page, a summary of the run is also given: query term and options chosen, number of genes and publications processed, as well as a hyperlink to download the complete results in the comma-separated values (CSV) format. Although this file may be difficult to interpret by humans, it can be used as input for other computational analysis tools. For example, we have implemented a "send data to Functional Enrichment Analysis" link in the result page (Figure 3) of GLAD4U for submitting a gene list to the functional enrichment analysis tool WebGestalt [49,50]. This function is particularly handy for the functional interpretation of a gene list, e.g., a list returned by a disease term query. It could help revealing biological processes associated with the disease. As an example, enrichment analysis on the first 100 genes returned by the "Obesity" query linked this disease to biological processes such as "fat cell differentiation" (20 genes, multiple-test adjusted enrichment p-value (adjp) = 5.27e-28), "lipid metabolic process" (39 genes, adjp = 5.05e-20) and "response to insulin stimulus" (17 genes, adjp = 4.99e-18). In addition, we have also implemented a "visualize genes in a protein-protein interaction network" link, which allows the visualization of interactions among the protein products of the genes based on the Cytoscape Web utility (http://cytoscapeweb.cytoscape.org/).

## Discussion

Reading through all relevant literature to generate a gene list is time consuming [10,51-53], a common concern that came up in all interviews of experimentalists that we performed (results not shown). GLAD4U addresses this problem by automatically creating a ranked list of genes following a user's input query.

One important feature of GLAD4U is its information processing. Based on our survey among experimentalists, GLAD4U follows the exact same steps that an experimentalist would follow: gather literature, extract gene information and create an expert list [54]. Whether a user queries a disease, a non-disease phenotype, a biological process or a gene, GLAD4U will fetch corresponding biomedical publications using NCBI's eUtilities API, retrieve relevant gene information, rank them and send them back to the user. GLAD4U ensures computational efficiency through effective use of existing NCBI resources, which also made it one of the winning applications in the National Library of Medicine (NLM)'s 2011 Software Development Challenge on the Innovative Uses of NLM Information.

Another important feature of GLAD4U is its simplicity. Researchers will be at ease using GLAD4U because its searching engine is powered by PubMed's API [48,52], and behaves similarly to Entrez-PubMed [55]. GLAD4U outputs a clean result page where the user can easily find genes relevant to the concept queried and supporting publications. Additionally, the use of PubMed's API makes GLAD4U almost maintenance-free. GLAD4U will update itself along with the MEDLINE library update. This will ensure that GLAD4U's results will always be up-to-date with the current literature.

Several tools rely on PubMed to build disease candidate genes lists [5,8,52,56,57]. EBIMed [5] and FACTA [7] are concept-oriented applications for mining existing biomedical literature. They attempt to automatically establish the publication-concept (including genes) relationship through in-house text mining tools whereas GLAD4U relies on the manually curated publication-gene mapping provided by NCBI. According to our results, manual mapping seems to have notable impact on performance. Nevertheless, automated mapping would allow flexibility in extending the services for concepts other than genes.

Although using the biomedical literature as a knowledge source seems intuitive [51,58,59], certain limitations exist: the literature is indexed based on titles, abstracts and keywords, not on full-text [60,61]. Thus, a set of publications retrieved may be incomplete (i.e., some publications relevant to the concept queried will not be retrieved because they do not contain the necessary keywords in their titles or abstracts) [62]. There is a possible bias in using the biomedical literature and ontology [55], as the most studied genes (those with the most publications) will have more weight [51,63] at the expense of more relevant genes that might only be featured in few papers [64]. Thus, we use the hypergeometric test to rank genes based on how likely it would be to retrieve them by chance alone, based on the number of publications retrieved for this gene among the total number of publications linked to this gene. The less likely it is--the smaller the *p *value--the higher the score will be for the gene. Thus, even if GLAD4U is solely retrieving its data from the biomedical literature, it prioritizes following a statistical analysis of the retrieved data.

The most obvious usage of GLAD4U is to generate a gene list for an input concept, which has been demonstrated in this paper. This can be extremely useful for the design of targeted high-throughput experiments. If one needs to create a custom array or selected proteins for targeted quantitative proteomic analysis using the selected reaction monitoring (SRM) assay, one can use GLAD4U and review the ranked list of genes that likely should be included in the experimental design. Besides generating gene lists for individual concepts, GLAD4U is very flexible and allows production of gene lists related to multiple concepts, which cannot be done by searching GO or OMIM databases. For example, a query of "smoking AND cancer" can generate a gene list that could potentially help exploring gene-environment interactions in cancer. GLAD4U also holds the potential to assist in improvement of the functional annotation of genes. Although GO contains more than 17,000 terms [4,65] and is regularly used in the bioinformatics field as a standard [4,66], it is not complete [51,67]. Through manual checking of the top genes returned by GLAD4U that were not part of the gold standard lists, we easily found evidence that these genes were indeed linked to the query, and probably should have been included in the gold standard.

Finally, because GLAD4U prioritization algorithm assigns scores to genes, removing the genes with a low score consistantly improves the quality of the results. This result justifies thresholding GLAD4U results by default.

## Conclusions

GLAD4U is a freely available web-application for creating expert candidate gene lists tailored to a user's query. It follows the same steps that the experimentalist would follow: gather literature, extract gene information and create an expert list. The simple interface of GLAD4U ensures easy usage and interpretation. Because GLAD4U relies on existing biomedical literature, it has an immediate credibility with experimentalists, who use this resource as a primary means for enhancing their knowledge and expertise. Although the gene list directly returned from a PubMed query is usually lengthy and noisy, the prioritization method implemented in GLAD4U successfully ranks truly relevant genes at the top of the list and facilitates efficient browsing of the list.

## Methods

### Publication retrieval

GLAD4U relies on the eSearch application programming interface (API) developed by the NCBI for retrieving publications from the MEDLINE database [48]. For a user query, eSearch returns an XML file containing the number of publications returned by the query and all publication identification IDs (PMIDs). The XML file is parsed to get the list of PMIDs associated with a user query.

### Gene retrieval

Genes associated with PMIDs are retrieved based on the gene-to-publication link table provided by Entrez-Gene [68]. Links between Entrez-Gene IDs and PMIDs are created based on both manual curation within the NCBI and integration of information from other public databases. Publications linked to more than 500 genes are removed from the link table because they lack specificity. After this process, the link table included 3,509,732 genes and 647,523 publications for all organisms, among which 30,343 genes and 306,487 publications were related to human (as of 05/14/2011).

### Gene prioritization

We studied two methods to prioritize the retrieved genes based on publication counts or the hypergeometric test. To prioritize using counts ("GLAD4U Counts"), each gene receives a score equal to the number of publications describing it in the link table. The other method ("GLAD4U Hypergeometric") uses the hypergeometric test to prioritize all retrieved genes. Specifically, for a given query Q and a gene G, let *n *be the number of publications retrieved for the query and present in the gene-to-publication link table (query-relevant publications) and *k *be the number of query-relevant publications that involves the gene G. Let us further assume that there are *m *publications in the gene-to-publication link table, *j *of which involve the gene G (gene-relevant publications). This method calculates the probability of observing *k *or more query-relevant publications for the gene by chance, based on the hypergeometric test and scores the gene using the following formula:

$S_{G}=-{\text{log}}_{10}^{f\left(m,n,j,k\right)}$, where

$$f_{\left(m,n,j,k\right)}=\overset{\text{min}\left(n,j\right)}{\underset{i=k}{\sum }}\frac{\left(\begin{array}{c} m-j\\ n-i \end{array}\right)\left(\begin{array}{c} j\\ i \end{array}\right)}{\left(\begin{array}{c} m\\ n \end{array}\right)}$$

### Performance evaluation

We used GO and disease terms as queries to evaluate the performance of the GLAD4U algorithms. Gene lists curated in GO, OMIM and GAD [69] were used as a gold standard (i.e. relevant genes). We developed a Perl script to parse the files "gene2go.gz" [68] and "gene_ontology.1_2.obo" [70] in order to generate gene lists for GO terms (as of 12/20/2009). Because of the parent-child relationship among the GO terms as described in the GO Direct Acyclic Graph, genes with granular annotations were associated with their parent terms using the Perl script. Using GAD, we identified all genes associated to a disease term. Using OMIM, we retrieved all IDs prefixed with "%" and "#" with the query in the title. Corresponding gene IDs were mapped by parsing the file "mim2gene" [68] (as of 12/22/2009). For each disease term, the lists obtained with GAD and OMIM were merged to serve as a gold standard. Retrieval performance was evaluated using precision, recall and F-measure. The F-measure is calculated by 2*pr*/(*p*+*r*), where *p *is the precision defined as $\left|\left\{relevant\;genes\right\}\cap \left\{retrieved\;genes\right\}\right|/\left|\left\{retrieved\;genes\right\}\right|$ and *r *is the recall defined as $\left|\left\{relevant\;genes\right\}\cap \left\{retrieved\;genes\right\}\right|/\left|\left\{relevant\;genes\right\}\right|$. We used the precision/recall curve, average precision (AP) and precision at the top *k *retrieved genes (*k *= 50 and *k *= 100) to evaluate the performance of our gene prioritization methods, and compared it to the performance of the ranked lists generated by EBIMed [6]. All performance values are expressed in the text as mean ± standard deviation.

### Web implementation

The GLAD4U user interface was developed in HTML and PHP languages. The scripts to deploy and update the algorithm on web servers were written in Perl, while the generation of hypergeometric test scores is using C. JQuery was used to implement user-features such as the ability to hide/ show options and functions. An email notification module was implemented to allow users to retrieve their results at a later time. GLAD4U (http://bioinfo.vanderbilt.edu/glad4u) is platform-independent and under a GNU GPL license [71]. It was tested on Internet Explorer 5.0, Firefox 3.0, Safari 3.0, Chrome, Netscape 7 or any higher versions of these browsers.

## List of abbreviations used

ADIPOQ: adiponectin; API: application programming interface; CSV: comma-separated values; GAD: genetic association database; GLAD4U Counts: GLAD4U prioritization algorithm using counts; GLAD4U Hypergeometric: GLAD4U prioritization algorithm using the hypergeometric test; GLAD4U: gene list automatically derived for you; GO: gene ontology; GOTM: GOTree Machine; ING1: inhibitor of growth family: member 1; AP: average precision; NCBI: national center for biotechnology information; OMIM: online mendelian inheritance in man; PMIDs: publication identification IDs; REN: renin; SRM: selected reaction monitoring.

## Competing interests

The authors declare that they have no competing interests.

## Authors' contributions

BZ and JJ conceived of the study, which was coordinated by BZ. JJ carried out the work with PHP and Perl, DD implemented the C version of the algorithm, ZS implemented the cytoscape web plugin for network visualization. JJ, DD, ZS and BZ participated in testing. JJ and BZ participated in the analysis of the results and in writing of the manuscript.

## Supplementary Material

Additional file 1 **False-positive genes retrieved by querying "apoptosis" with GLAD4U**. This table shows all genes retrieved by GLAD4U with the query "apoptosis" that were not among the gold standards. The table presents the rank and score of these genes and all the retrieved supporting publications. Click here for file

Additional file 2 **False-positive genes retrieved by querying "hypertension" with GLAD4U**. This table shows all genes retrieved by GLAD4U with the query "hypertension" that were not among the gold standards. The table presents the rank and score of these genes and all the retrieved supporting publications. Click here for file

Additional file 3 **GLAD4U prioritization of disease candidate genes**. This table shows the number of genes associated with each GeneWanderer hereditaty disease, retrieved by GLAD4U and overlapping between the two lists before and after thresholding. F-measure fold change between the GLAD4U prioritized list before and after thresholding, as well as the actual F-measures are also displayed in the table. Click here for file

## References

[B1] Ashburner M Ball CA Blake JA Botstein D Butler H Cherry JM Davis AP Dolinski K Dwight SS Eppig JT Gene ontology: tool for the unification of biology. The Gene Ontology Consortium Nat Genet 2000 25 1 25 29 10.1038/75556 10802651 PMC3037419

[B2] The Gene Ontology http://www.geneontology.org/

[B3] Online Mendelian Inheritance in Man http://www.ncbi.nlm.nih.gov/omim/

[B4] Erhardt RA Schneider R Blaschke C Status of text-mining techniques applied to biomedical text Drug Discov Today 2006 11 7-8 315 325 10.1016/j.drudis.2006.02.011 16580973

[B5] Rebholz-Schuhmann D Kirsch H Arregui M Gaudan S Riethoven M Stoehr P EBIMed--text crunching to gather facts for proteins from Medline Bioinformatics 2007 23 2 e237 244 10.1093/bioinformatics/btl302 17237098

[B6] EBIMed http://www.ebi.ac.uk/Rebholz-srv/ebimed/index.jsp

[B7] Tsuruoka Y Tsujii J Ananiadou S FACTA: a text search engine for finding associated biomedical concepts Bioinformatics 2008 24 21 2559 2560 10.1093/bioinformatics/btn469 18772154 PMC2572701

[B8] Hur J Schuyler AD States DJ Feldman EL SciMiner: web-based literature mining tool for target identification and functional enrichment analysis Bioinformatics 2009 25 6 838 840 10.1093/bioinformatics/btp049 19188191 PMC2654801

[B9] GAD http://geneticassociationdb.nih.gov/

[B10] Becker KG Hosack DA Dennis G Lempicki RA Bright TJ Cheadle C Engel J PubMatrix: a tool for multiplex literature mining BMC Bioinformatics 2003 4 61 10.1186/1471-2105-4-61 14667255 PMC317283

[B11] Tanabe L Scherf U Smith LH Lee JK Hunter L Weinstein JN MedMiner: an Internet text-mining tool for biomedical information, with application to gene expression profiling Biotechniques 1999 27 6 1210 1214 1216-1217 10631500 10.2144/99276bc03

[B12] Castera L Sabbagh A Dehainault C Michaux D Mansuet-Lupo A Patillon B Lamar E Aerts I Lumbroso-Le Rouic L Couturier J MDM2 as a modifier gene in retinoblastoma J Natl Cancer Inst 2010 102 23 1805 1808 10.1093/jnci/djq416 21051655

[B13] Nardinocchi L Puca R Givol D D'Orazi G Counteracting MDM2-induced HIPK2 downregulation restores HIPK2/p53 apoptotic signaling in cancer cells FEBS Lett 2010 584 19 4253 4258 10.1016/j.febslet.2010.09.018 20849851

[B14] Post SM Quintas-Cardama A Pant V Iwakuma T Hamir A Jackson JG Maccio DR Bond GL Johnson DG Levine AJ A high-frequency regulatory polymorphism in the p53 pathway accelerates tumor development Cancer Cell 2010 18 3 220 230 10.1016/j.ccr.2010.07.010 20832750 PMC2944041

[B15] Yan J Di Y Shi H Rao H Huo K Overexpression of SCYL1-BP1 stabilizes functional p53 by suppressing MDM2-mediated ubiquitination FEBS Lett 2010 584 20 4319 4324 10.1016/j.febslet.2010.09.019 20849854 PMC3789512

[B16] Phillips A Teunisse A Lam S Lodder K Darley M Emaduddin M Wolf A Richter J de Lange J Verlaan-de Vries M HDMX-L is expressed from a functional p53-responsive promoter in the first intron of the HDMX gene and participates in an autoregulatory feedback loop to control p53 activity J Biol Chem 2010 285 38 29111 29127 10.1074/jbc.M110.129726 20659896 PMC2937942

[B17] Lai KP Leong WF Chau JF Jia D Zeng L Liu H He L Hao A Zhang H Meek D S6K1 is a multifaceted regulator of Mdm2 that connects nutrient status and DNA damage response EMBO J 2010 29 17 2994 3006 10.1038/emboj.2010.166 20657550 PMC2944047

[B18] Mandal RK Mittal RD Are cell cycle and apoptosis genes associated with prostate cancer risk in North Indian population? Urol Oncol 2012 10.1016/j.urolonc.2010.05.006 20822933

[B19] Gordon PM Soliman MA Bose P Trinh Q Sensen CW Riabowol K Interspecies data mining to predict novel ING-protein interactions in human BMC Genomics 2008 9 426 10.1186/1471-2164-9-426 18801192 PMC2565686

[B20] Garate M Wong RP Campos EI Wang Y Li G NAD(P)H quinone oxidoreductase 1 inhibits the proteasomal degradation of the tumour suppressor p33(ING1b) EMBO Rep 2008 9 6 576 581 10.1038/embor.2008.48 18388957 PMC2427386

[B21] Kuo WH Wang Y Wong RP Campos EI Li G The ING1b tumor suppressor facilitates nucleotide excision repair by promoting chromatin accessibility to XPA Exp Cell Res 2007 313 8 1628 1638 10.1016/j.yexcr.2007.02.010 17379210

[B22] Russell MW Soliman MA Schriemer D Riabowol K ING1 protein targeting to the nucleus by karyopherins is necessary for activation of p21 Biochem Biophys Res Commun 2008 374 3 490 495 10.1016/j.bbrc.2008.07.076 18655775

[B23] Garate M Campos EI Bush JA Xiao H Li G Phosphorylation of the tumor suppressor p33(ING1b) at Ser-126 influences its protein stability and proliferation of melanoma cells FASEB J 2007 21 13 3705 3716 10.1096/fj.07-8069com 17585055

[B24] Soliman MA Berardi P Pastyryeva S Bonnefin P Feng X Colina A Young D Riabowol K ING1a expression increases during replicative senescence and induces a senescent phenotype Aging Cell 2008 7 6 783 794 10.1111/j.1474-9726.2008.00427.x 18691180

[B25] Zhu Z Luo Z Li Y Ni C Li H Zhu M Human inhibitor of growth 1 inhibits hepatoma cell growth and influences p53 stability in a variant-dependent manner Hepatology 2009 49 2 504 512 10.1002/hep.22675 19085961

[B26] Han X Feng X Rattner JB Smith H Bose P Suzuki K Soliman MA Scott MS Burke BE Riabowol K Tethering by lamin A stabilizes and targets the ING1 tumour suppressor Nat Cell Biol 2008 10 11 1333 1340 10.1038/ncb1792 18836436

[B27] Gonzalez L Freije JM Cal S Lopez-Otin C Serrano M Palmero I A functional link between the tumour suppressors ARF and p33ING1 Oncogene 2006 25 37 5173 5179 16607280 10.1038/sj.onc.1209526

[B28] Pena PV Hom RA Hung T Lin H Kuo AJ Wong RP Subach OM Champagne KS Zhao R Verkhusha VV Histone H3K4me3 binding is required for the DNA repair and apoptotic activities of ING1 tumor suppressor J Mol Biol 2008 380 2 303 312 10.1016/j.jmb.2008.04.061 18533182 PMC2576750

[B29] Vefring HK Wee L Jugessur A Gjessing HK Nilsen ST Lie RT Maternal angiotensinogen (AGT) haplotypes, fetal renin (REN) haplotypes and risk of preeclampsia; estimation of gene-gene interaction from family-triad data BMC Med Genet 2010 11 90 20537141 10.1186/1471-2350-11-90 PMC2901215

[B30] Irvin MR Lynch AI Kabagambe EK Tiwari HK Barzilay JI Eckfeldt JH Boerwinkle E Davis BR Ford CE Arnett DK Pharmacogenetic association of hypertension candidate genes with fasting glucose in the GenHAT Study J Hypertens 2010 28 10 2076 2083 20577119 10.1097/HJH.0b013e32833c7a4d PMC2957368

[B31] Vangjeli C Clarke N Quinn U Dicker P Tighe O Ho C O'Brien E Stanton AV Confirmation that the renin gene distal enhancer polymorphism REN-5312C/T is associated with increased blood pressure Circ Cardiovasc Genet 2010 3 1 53 59 10.1161/CIRCGENETICS.109.899930 20160196

[B32] Ehret GB O'Connor AA Weder A Cooper RS Chakravarti A Follow-up of a major linkage peak on chromosome 1 reveals suggestive QTLs associated with essential hypertension: GenNet study Eur J Hum Genet 2009 17 12 1650 1657 10.1038/ejhg.2009.94 19536175 PMC2783544

[B33] Radi ZA Murad Y Cellular expression of renal, cardiac and pulmonary inducible nitric oxide synthase in double-transgenic mice expressing human renin and angiotensinogen genes Clin Exp Pharmacol Physiol 2009 36 5-6 571 575 10.1111/j.1440-1681.2008.05120.x 19673942

[B34] Biala A Tauriainen E Siltanen A Shi J Merasto S Louhelainen M Martonen E Finckenberg P Muller DN Mervaala E Resveratrol induces mitochondrial biogenesis and ameliorates Ang II-induced cardiac remodeling in transgenic rats harboring human renin and angiotensinogen genes Blood Press 2010 19 3 196 205 10.3109/08037051.2010.481808 20429690

[B35] Celoria BM Genelhu VA Pimentel Duarte SF Delfraro PA Francischetti EA Hypoadiponectinemia is associated with prehypertension in obese individuals of multiethnic origin Clin Cardiol 2010 33 6 E61 65 10.1002/clc.20657 20552610 PMC6653684

[B36] Paakko T Ukkola O Ikaheimo M Kesaniemi YA Plasma adiponectin levels are associated with left ventricular hypertrophy in a random sample of middle-aged subjects Ann Med 2010 42 2 131 137 20166815 10.3109/07853890903449827

[B37] Elenkova A Matrozova J Zacharieva S Kirilov G Kalinov K Adiponectin - A possible factor in the pathogenesis of carbohydrate metabolism disturbances in patients with pheochromocytoma Cytokine 2010 50 3 306 310 10.1016/j.cyto.2010.03.011 20385503

[B38] Shim CY Park S Kim JS Shin DJ Ko YG Kang SM Choi D Ha JW Jang Y Chung N Association of plasma retinol-binding protein 4, adiponectin, and high molecular weight adiponectin with insulin resistance in non-diabetic hypertensive patients Yonsei Med J 2010 51 3 375 384 10.3349/ymj.2010.51.3.375 20376890 PMC2852793

[B39] Ix JH Sharma K Mechanisms linking obesity, chronic kidney disease, and fatty liver disease: the roles of fetuin-A, adiponectin, and AMPK J Am Soc Nephrol 2010 21 3 406 412 10.1681/ASN.2009080820 20150538 PMC4473254

[B40] Persson J Lindberg K Gustafsson TP Eriksson P Paulsson-Berne G Lundman P Low plasma adiponectin concentration is associated with myocardial infarction in young individuals J Intern Med 2010 268 2 194 205 10.1111/j.1365-2796.2010.02247.x 20528971

[B41] Leu HB Chung CM Chuang SY Bai CH Chen JR Chen JW Pan WH Genetic variants of connexin37 are associated with carotid intima-medial thickness and future onset of ischemic stroke Atherosclerosis 2011 214 1 101 106 10.1016/j.atherosclerosis.2010.10.010 21044781

[B42] Wilke RA Simpson RU Mukesh BN Bhupathi SV Dart RA Ghebranious NR McCarty CA Genetic variation in CYP27B1 is associated with congestive heart failure in patients with hypertension Pharmacogenomics 2009 10 11 1789 1797 10.2217/pgs.09.101 19891555 PMC2974901

[B43] Niu W Qi Y Guo S Gao P Zhu D Association of renin BglI polymphism with essential hypertension: a meta-analysis involving 1811 cases and 1626 controls Clin Exp Hypertens 2010 32 7 431 438 10.3109/10641961003686419 20925572

[B44] Ying CQ Wang YH Wu ZL Fang MW Wang J Li YS Zhang YH Qiu CC Association of the renin gene polymorphism, three angiotensinogen gene polymorphisms and the haplotypes with essential hypertension in the Mongolian population Clin Exp Hypertens 2010 32 5 293 300 10.3109/10641960903443517 20662730

[B45] Ragia G Nikolaidis E Tavridou A Arvanitidis KI Kanoni S Dedoussis GV Bougioukas G Manolopoulos VG Renin-angiotensin-aldosterone system gene polymorphisms in coronary artery bypass graft surgery patients J Renin Angiotensin Aldosterone Syst 2010 11 2 136 145 10.1177/1470320310361742 20223792

[B46] Ong KL Li M Tso AW Xu A Cherny SS Sham PC Tse HF Lam TH Cheung BM Lam KS Association of genetic variants in the adiponectin gene with adiponectin level and hypertension in Hong Kong Chinese Eur J Endocrinol 2010 163 2 251 257 10.1530/EJE-10-0251 20516205

[B47] Kohler S Bauer S Horn D Robinson PN Walking the interactome for prioritization of candidate disease genes Am J Hum Genet 2008 82 4 949 958 10.1016/j.ajhg.2008.02.013 18371930 PMC2427257

[B48] Masys DR Linking microarray data to the literature Nat Genet 2001 28 1 9 10 11326264 10.1038/ng0501-9

[B49] Zhang B Schmoyer D Kirov S Snoddy J GOTree Machine (GOTM): a web-based platform for interpreting sets of interesting genes using Gene Ontology hierarchies BMC Bioinformatics 2004 5 16 10.1186/1471-2105-5-16 14975175 PMC373441

[B50] WebGestalt (WEB-based GEne SeT AnaLysis Toolkit) http://bioinfo.vanderbilt.edu/webgestalt/10.1093/nar/gkt439 PMC3692109 23703215

[B51] Aerts S Lambrechts D Maity S Van Loo P Coessens B De Smet F Tranchevent LC De Moor B Marynen P Hassan B Gene prioritization through genomic data fusion Nat Biotechnol 2006 24 5 537 544 10.1038/nbt1203 16680138

[B52] Cheng D Knox C Young N Stothard P Damaraju S Wishart DS PolySearch: a web-based text mining system for extracting relationships between human diseases, genes, mutations, drugs and metabolites Nucleic Acids Res 2008 36 Web Server issue W399 405 18487273 10.1093/nar/gkn296 PMC2447794

[B53] Zhang B Kirov S Snoddy J WebGestalt: an integrated system for exploring gene sets in various biological contexts Nucleic Acids Res 2005 33 Web Server issue W741 748 15980575 10.1093/nar/gki475 PMC1160236

[B54] Khatri P Bhavsar P Bawa G Draghici S Onto-Tools: an ensemble of web-accessible, ontology-based tools for the functional design and interpretation of high-throughput gene expression experiments Nucleic Acids Res 2004 32 Web Server issue W449 456 15215428 10.1093/nar/gkh409 PMC441547

[B55] Jensen LJ Saric J Bork P Literature mining for the biologist: from information retrieval to biological discovery Nat Rev Genet 2006 7 2 119 129 10.1038/nrg1768 16418747

[B56] Chen J Xu H Aronow BJ Jegga AG Improved human disease candidate gene prioritization using mouse phenotype BMC Bioinformatics 2007 8 392 10.1186/1471-2105-8-392 17939863 PMC2194797

[B57] Plake C Schiemann T Pankalla M Hakenberg J Leser U AliBaba: PubMed as a graph Bioinformatics 2006 22 19 2444 2445 10.1093/bioinformatics/btl408 16870931

[B58] de Bruijn DR dos Santos NR Kater-Baats E Thijssen J van den Berk L Stap J Balemans M Schepens M Merkx G van Kessel AG The cancer-related protein SSX2 interacts with the human homologue of a Ras-like GTPase interactor, RAB3IP, and a novel nuclear protein, SSX2IP Genes Chromosomes Cancer 2002 34 3 285 298 10.1002/gcc.10073 12007189

[B59] Turner FS Clutterbuck DR Semple CA POCUS: mining genomic sequence annotation to predict disease genes Genome Biol 2003 4 11 R75 10.1186/gb-2003-4-11-r75 14611661 PMC329128

[B60] Jenssen TK Laegreid A Komorowski J Hovig E A literature network of human genes for high-throughput analysis of gene expression Nat Genet 2001 28 1 21 28 11326270 10.1038/ng0501-21

[B61] Muller HM Kenny EE Sternberg PW Textpresso: an ontology-based information retrieval and extraction system for biological literature PLoS Biol 2004 2 11 e309 10.1371/journal.pbio.0020309 15383839 PMC517822

[B62] Grivell L Mining the bibliome: searching for a needle in a haystack? New computing tools are needed to effectively scan the growing amount of scientific literature for useful information EMBO Rep 2002 3 3 200 203 10.1093/embo-reports/kvf059 11882534 PMC1084023

[B63] Tiffin N Adie E Turner F Brunner HG van Driel MA Oti M Lopez-Bigas N Ouzounis C Perez-Iratxeta C Andrade-Navarro MA Computational disease gene identification: a concert of methods prioritizes type 2 diabetes and obesity candidate genes Nucleic Acids Res 2006 34 10 3067 3081 10.1093/nar/gkl381 16757574 PMC1475747

[B64] Perez-Iratxeta C Bork P Andrade MA Association of genes to genetically inherited diseases using data mining Nat Genet 2002 31 3 316 319 12006977 10.1038/ng895

[B65] Bada M Stevens R Goble C Gil Y Ashburner M Blake JA Cherry JM Harris M Lewis S A short study on the success of the Gene Ontology Web Semantics: Science, Services and Agents on the World Wide Web 2004 1 235 240 10.1016/j.websem.2003.12.003

[B66] Tiffin N Kelso JF Powell AR Pan H Bajic VB Hide WA Integration of text- and data-mining using ontologies successfully selects disease gene candidates Nucleic Acids Res 2005 33 5 1544 1552 10.1093/nar/gki296 15767279 PMC1065256

[B67] Wren JD Garner HR Shared relationship analysis: ranking set cohesion and commonalities within a literature-derived relationship network Bioinformatics 2004 20 2 191 198 10.1093/bioinformatics/btg390 14734310

[B68] Entrez Gene FTP ftp://ftp.ncbi.nih.gov/gene/DATA/

[B69] Becker KG Barnes KC Bright TJ Wang SA The genetic association database Nat Genet 2004 36 5 431 432 10.1038/ng0504-431 15118671

[B70] Gene Ontology OBO data http://geneontology.org/ontology/obo_format_1_2/

[B71] GNU GPL http://www.gnu.org/licenses/#GPL

